# HPV Vaccination Uptake Among Vaccine-Eligible U.S. Adults with and Without a Cancer History: A National Health and Nutrition Examination Survey Analysis, 2011–2023

**DOI:** 10.3390/vaccines14100892

**Published:** 2026-10-12

**Authors:** Jessica Saintibert, Tammie M. Johnson

**Affiliations:** College of Pharmacy and Pharmaceutical Sciences, Institute of Public Health, Florida A&M University, Tallahassee, FL 32307, USA; jessica1.saintibert@famu.edu

**Keywords:** human papillomavirus, human papillomavirus adult vaccination, National Health and Nutrition Examination Survey, human papillomavirus-associated cancers, cancer prevention

## Abstract

Background: Human papillomavirus includes more than 200 types, classified by cancer risk. Since 2006, the vaccine has effectively prevented human papillomavirus infection and associated cancers; however, coverage among adults remains low. Furthermore, research examining human papillomavirus vaccination among adults with a cancer history is limited. Objective: To estimate vaccination uptake for human papillomavirus among U.S. adults aged 20–47 and compare uptake by cancer history. Methods: This study used data from the National Health and Nutrition Examination Survey from 2011 to 2023. The final analytic sample included 13,511 adults aged 20–47 with self-reported vaccination status and cancer history. The data were weighted to account for the complex survey design and produce nationally representative estimates. Results: Among eligible adults, 84.7% were unvaccinated, 2.9% were under-vaccinated, and 12.4% had completed the vaccine series. Among eligible adults with a self-reported cancer history, the majority were unvaccinated. Vaccination completion differed by sociodemographic and healthcare-related factors. Conclusions: Human papillomavirus vaccination remained low among eligible U.S. adults, including those with a self-reported cancer history. These findings support efforts to improve adult vaccination coverage and prevent human papillomavirus infections and associated cancers.

## 1. Introduction

Human papillomavirus (HPV) is a non-enveloped, double-stranded DNA virus with more than 200 types, classified as low-risk (non-oncogenic) or high-risk (oncogenic) [1,2]. In the United States (U.S.), the estimated annual incidence and prevalence are approximately 13 million and 42 million infections, respectively [3]. Transmission can occur through skin-to-skin contact, mucosa-to-mucosa, autoinoculation, and vertical transmission from mother to child, with some, if not most, individuals remaining asymptomatic [4,5,6,7]. Progression from infection to cancer occurs primarily through persistent infection, defined as the repeated presence of the same HPV type across multiple visits over a period of time [8,9,10]. HPV has been detected in cancers of the cervix, anus, penis, vulva, vagina, oral cavity, larynx, and oropharynx [1,8,11,12,13,14,15]. From 2012 to 2016, an estimated 43,999 HPV-associated cancers occurred annually in the U.S., with 79% attributable to HPV and 92% caused by vaccine-targeted types, with oropharyngeal cancer accounting for the largest proportion from 2014 to 2018 [16,17].

HPV is an important cause of the increasing incidence of oral and oropharyngeal squamous cell carcinoma and other associated cancers, with disparities in disease burden and multiple risk factors, including tobacco and alcohol [18,19,20,21,22]. HPV has additionally been investigated in colorectal and esophageal cancers, although findings remain inconsistent and causality is unconfirmed [23,24,25,26,27,28,29,30,31,32,33,34,35]. Cervical cancer is largely preventable and predominantly caused by HPV, particularly types 16 and 18, yet it continues to cause over 4000 U.S. deaths annually, with persistent racial disparities in survival and mortality [36,37,38,39,40]. HPV DNA has also been detected in some ovarian and uterine cancers, although findings remain inconsistent [41,42,43,44,45,46,47]. Associations between HPV and prostate and testicular cancers remain uncertain. HPV has been detected in prostate tissues and semen, but current evidence does not establish a causal relationship [48,49,50,51,52,53,54,55,56].

The most effective preventive strategy against HPV-associated cancers and infections is Gardasil-9, the only FDA-approved HPV vaccine since 2016–2017, which protects against nine HPV types [57,58]. The HPV vaccine was first approved in 2006 for girls and young women aged 9–26 and was later expanded to include males and adults up to 45 [57,58,59,60,61]. Based on guidelines, if the first dose was received before the 15th birthday, then the recommendation is two doses; however, if the first dose is given on the 15th birthday or later, then the recommendation is three doses [60]. Despite its effectiveness, vaccination coverage remains low, with the female population having higher uptake than the male population [62,63,64]. Low HPV vaccination uptake has also been reported among cancer survivors [65,66,67].

Previous studies demonstrated that HPV vaccination uptake and series completion vary based on age, gender, demographic, socioeconomic, and healthcare-related determinants among adolescents and adults [15,18,62,63,68,69]. Socioeconomic determinants, including race/ethnicity, education, income, and marital status, have also been associated with HPV vaccination initiation, uptake, or series completion [70,71,72,73,74,75]. Meanwhile, healthcare-related determinants, includes health insurance, healthcare access, communication with healthcare providers, and financial barriers to care [70,72,73,76,77,78,79].

Much of the HPV vaccination literature has focused on adolescents and young adults through age 26, while other studies have examined vaccination uptake and associated factors in the broader population rather than specifically among adults with a history of cancer [67,71,80]. Among cancer survivors, the available literature has largely centered on childhood to young adult cancer survivors or select cancer populations [65,81,82]. However, more recent studies have begun to examine HPV vaccination among adult cancer survivors, though evidence remains limited [65,66,67].

Thus, there remains a need to comprehensively assess HPV vaccine series completion among adults, including those with a history of cancer. Using the National Health and Nutrition Examination Survey (NHANES), the objective is to estimate the prevalence of the HPV vaccine series completion among U.S. adults aged 20–47 and examine differences by cancer history. The hypothesis is that adults with a history of HPV-associated cancers would demonstrate higher HPV vaccine uptake than adults without such histories. The aim is to inform public health strategies to increase uptake, address gender-based disparities, and reduce the preventable burden of HPV among adults [64,83,84,85,86,87].

## 2. Methods

### 2.1. Study Population

The study population included 34,048 U.S. adults aged 20 or older who participated in NHANES from 2011 to 2023 (Table 1). Adults younger than 20 were excluded because NHANES collects cancer history information beginning at age 20. The population was then restricted to adults aged 20–47 years (n = 15,117). Of these, 13,514 had reported their HPV vaccination status, and 13,511 reported their cancer history, resulting in a final analytical sample of 13,511 participants aged 20–47.

### 2.2. Study Variables

The primary outcome was any HPV vaccination based on self-report of ever receiving the HPV vaccine (yes/no). The number of doses received was included to define HPV vaccination completion, with participants reporting zero doses categorized as unvaccinated, one dose as under-vaccinated, and 2 or 3 doses as fully vaccinated. Fully vaccinated was defined based on guidelines from the Advisory Committee on Immunization Practices (ACIP) and the World Health Organization (WHO), which consider two and three doses as complete. This classification is supported by clinical trials demonstrating that adolescents younger than 15 who received two doses had immune responses non-inferior to those aged 15 or older who received three doses [88,89,90,91].

Vaccine eligibility was operationally defined based on the age range captured by NHANES HPV vaccination question of ever receiving the HPV vaccine and the possibility of prior HPV vaccination, rather than current age eligibility alone. The study population, therefore, included adults up to 47, as NHANES collected HPV vaccination history among adults up to age 49, extending beyond the current recommended age range [79,92]. HPV vaccination has also been evaluated among adults older than 45 in selected clinical populations, despite falling outside current U.S. licensing and ACIP recommendations [93,94]. The primary independent variable was self-reported cancer history, based on self-reported responses to whether a health professional had ever told the participant they had cancer (yes/no).

Participants’ self-reported cancer histories of interest were classified using three analytic approaches. First, cancers were categorized by sex specificity into three groups: non-gender-specific cancers (oral/oropharyngeal, colon, rectal (rectum), and esophageal), female-specific cancers (cervical, ovarian, and uterine), and male-specific cancers (prostate and testicular cancer). Cancers that affect both women and men were defined as non-gender-specific. Second, cancers were categorized according to their association with HPV: HPV-associated or cancers with uncertain HPV association with respect to HPV’s oncogenic properties (Supplementary Table S1). Third, the HPV-associated cancers and the cancers with uncertain HPV association were evaluated separately among participants aged 20 to 26 and 27 to 47 to assess the odds of being fully vaccinated against HPV (2 or 3 doses) across the age groups affected by the expansion of the recommended age range for HPV vaccination.

Additional covariates included income, education, health insurance, marital status, race/ethnicity, healthcare access, and gender. Covariates were selected based on their established or plausible associations with HPV vaccination and cancer history. Age, gender, race/ethnicity, and marital status were considered potential demographic confounders, while income and education represented socioeconomic factors, and health insurance and healthcare access represented healthcare-related factors that may influence HPV vaccination completion.

### 2.3. Statistical Analyses

Statistical analyses were conducted among the study population of U.S. adults aged 20–47, where SAS version 9.4 was used to conduct all analyses. Participants with responses of “missing,” “don’t know,” or “refused” for self-reported HPV vaccination, including the number of doses received, self-reported cancer history, cancer type, or other covariates included in the study, were recoded as missing and excluded from the analysis. NHANES weights, strata, and primary sampling units were incorporated to account for complex survey design, oversampling, and nonresponse and to produce nationally representative estimates. Weighted frequencies, percentages, and 95% confidence intervals (CI) were calculated using PROC SURVEYFREQ to summarize HPV vaccination status and participant characteristics.

Bivariate associations between HPV vaccination status and participant characteristics were assessed using Rao-Scott chi-square tests, with *p*-values obtained from the Rao-Scott chi-square test. Multivariable survey-weighted logistic regression models were conducted using PROC SURVEYLOGISTIC to evaluate associations between cancer history and the odds of being fully vaccinated, while adjusting for covariates. Domain analysis was used within the regression models to account for the complex survey design. Adjusted odds ratios and 95% CI were reported, with *p*-values for individual model effects obtained from Wald F tests. Statistical significance was defined as *p* < 0.05.

## 3. Results

The final analytic sample consisted of 13,511 U.S. adults aged 20–47, representing over 105.9 million U.S. adults. Most adults were in the 30–39 age group (n = 4988; weighted n = 37.7 million). The majority were non-Hispanic White, married or living with a partner. Approximately one-third had a college education or higher (32.9%) (Table 2).

**Figure 1:**
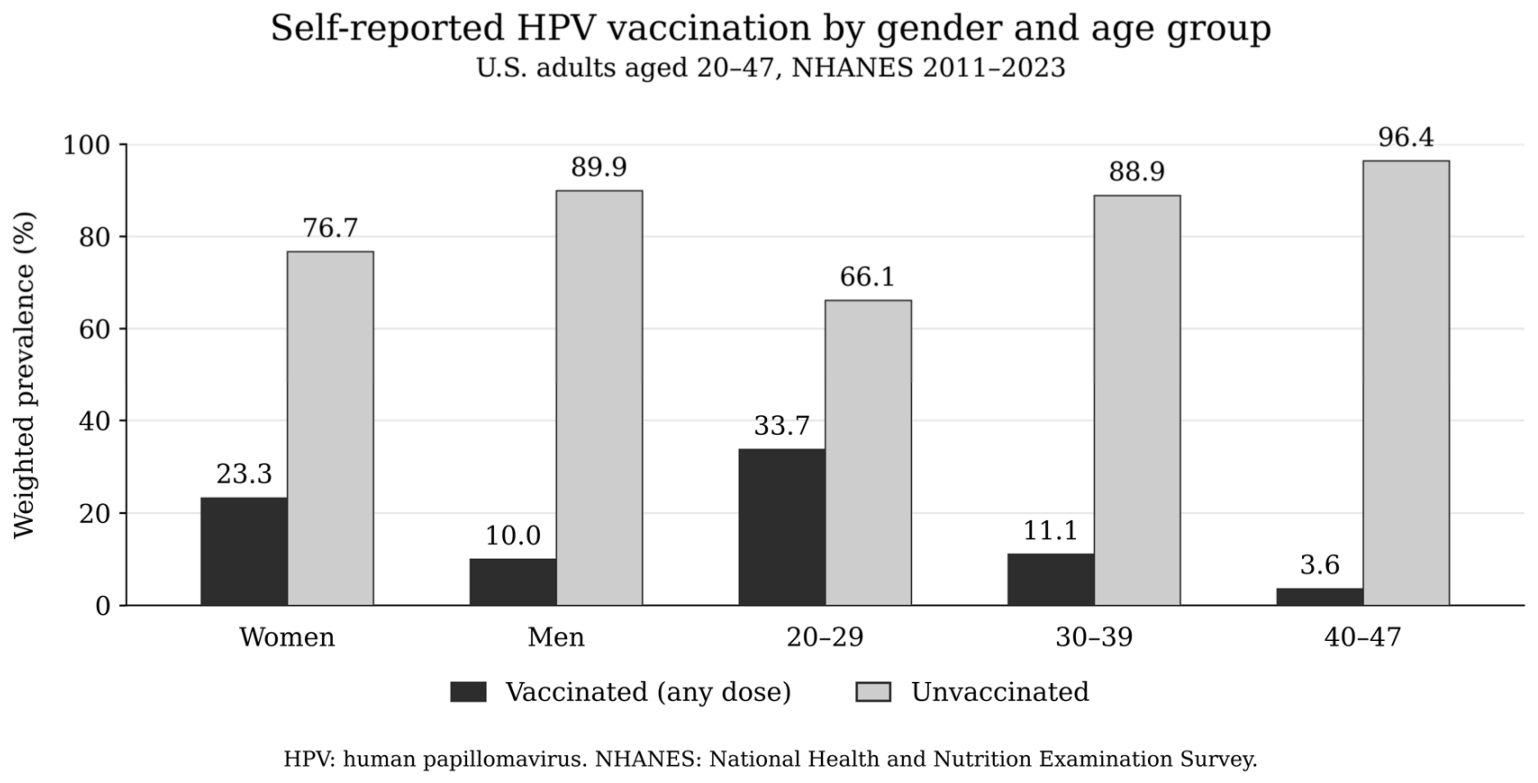
Weighted prevalence of self-reported HPV vaccination status by gender and age group among U.S. adults aged 20-47, NHANES 2011–2023.

Among adults who reported their HPV vaccination status, a large proportion of women and men were unvaccinated, representing approximately 43.5 million women and 46.3 million men. Any HPV vaccination prevalence was higher among women than men (23.3% vs. 10.0%, respectively). Any vaccination prevalence also decreased with increasing age, from 33.7% among adults aged 20–29 to 11.1% among those aged 30–39, and 3.6% among adults aged 40–47. These weighted prevalence estimates represent approximately 12.9 million adults aged 20–29, 4.2 million adults aged 30–39, and 1.1 million adults aged 40–47 (Figure 1).

Weighted bivariate analysis showed that gender (*p* < 0.0001), age (*p* < 0.0001), marital status (*p* < 0.0001), race/ethnicity (*p* < 0.0001), income (*p* = 0.038), education (*p* < 0.0001), healthcare access (*p* < 0.0001), health insurance (*p* < 0.0001), self-reported female-specific cancer history (*p* = 0.0003) were significantly associated with HPV vaccination completion. The number of HPV vaccine doses received was associated with gender (*p* < 0.0001), age (*p* = 0.0004), race/ethnicity (*p* < 0.0001), education (*p* < 0.0001), and health insurance (*p* = 0.0002). Participants who reported never receiving the HPV vaccine history were retained in the dose-based analyses and were categorized as zero doses. This conservative assumption allowed for a more comprehensive representation of vaccine uptake in the eligible adult population.

Among women, age group and self-reported female-specific cancer history were significantly associated with HPV vaccination completion. Women aged 27–47 had 83% lower odds of being fully vaccinated compared with women aged 20–26 (OR = 0.17; 95% CI: 0.14–0.20), while women without a self-reported female-specific cancer history had higher odds of being fully vaccinated than those with a history (OR = 3.17; 95% CI: 1.47–6.85). After adjustment for sociodemographic and healthcare-related factors, these associations remained significant (age group: OR = 0.16; 95% CI: 0.13–0.19; female-specific cancer history: OR = 2.73; 95% CI: 1.28–5.84). Race/ethnicity was significantly associated with HPV vaccination completion (*p* = 0.014). Compared with non-Hispanic White women, non-Hispanic Black women had 32% lower odds of being fully vaccinated (OR = 0.68; 95% CI: 0.54–0.87). No significant differences were observed for Hispanic, non-Hispanic Asian, or Other/Multiracial women. Health insurance, education, and marital status were also significantly associated with vaccination completion, whereas income and healthcare access were not significantly associated (Table 3).

Among men, age group was significantly associated with HPV vaccination completion, whereas self-reported male-specific cancer history was not significantly associated. Men aged 27–47 had 78% lower odds of being fully vaccinated compared with men aged 20–26 (OR = 0.22; 95% CI: 0.16–0.29). After adjustment for sociodemographic and healthcare-related factors, age remained significantly associated with vaccination completion (OR = 0.23; 95% CI: 0.15–0.34), while male-specific cancer history remained non-significant (OR = 0.23; 95% CI: 0.03–1.75). Health insurance and education were also significantly associated with vaccination completion. Race/ethnicity was not significantly associated with HPV vaccination completion (*p* = 0.133). However, compared with non-Hispanic White men, non-Hispanic Asian men had 51% lower odds of being fully vaccinated (OR = 0.49; 95% CI: 0.26–0.91). No significant differences were observed for Hispanic, non-Hispanic Black, or Other/Multiracial men. Marital status, income, and healthcare access were not significantly associated with vaccination completion (Table 4).

For adults with and without a self-reported non-gender-specific cancer history, age group was significantly associated with HPV vaccination completion, whereas non-gender-specific cancer history was not. Adults aged 27–47 had 81% lower odds of being fully vaccinated compared with adults aged 20–26 (OR = 0.19; 95% CI: 0.17–0.23). After adjustment for sociodemographic and healthcare-related factors, age remained significantly associated with vaccination completion (OR = 0.18; 95% CI: 0.15–0.22), while non-gender-specific cancer history remained non-significant (OR = 1.31; 95% CI: 0.81–2.13). Gender, health insurance, education, race/ethnicity, and marital status were also significantly associated with HPV vaccination completion. Women had higher odds of being fully vaccinated compared with men (OR = 4.21; 95% CI: 3.41–5.19). Compared with non-Hispanic White adults, non-Hispanic Asian and non-Hispanic Black adults each had 31% lower odds of being fully vaccinated (OR = 0.69; 95% CI: 0.56–0.87 and OR = 0.69; 95% CI: 0.56–0.86, respectively). No significant differences were observed for Hispanic or Other/Multiracial adults; income and healthcare access were also not significantly associated with vaccination completion (Table 5).

Descriptive analyses provided further detail on HPV vaccination completion according to self-reported female-specific, male-specific, and non-gender-specific cancer history. Vaccination completion was 5.3% among women with a female-specific cancer history compared with 18.6% among those without, 16.5% among men with a male-specific cancer history compared with 5.7% among those without, and 16.5% among adults with a non-gender-specific cancer history compared with 12.4% among those without (Supplementary Tables S2–S4). Additional regression analyses examined HPV vaccination completion according to self-reported overall cancer history, self-reported HPV-associated cancer history, and self-reported history of cancers with uncertain HPV association. None of the cancer history measures were significantly associated with HPV vaccination completion. Age remained significantly associated with HPV vaccination completion across all three cancer history analyses, including when evaluated by age group (Supplementary Tables S5–S7).

Age-stratified analyses compared adults aged 20–26 and 27–47 to reflect the expansion of the recommended age range for HPV vaccination. Adults aged 27–47 had lower odds of being fully vaccinated than adults aged 20–26 (OR = 0.19; 95% CI: 0.17–0.23) (Supplementary Table S8). Age group remained significantly associated with vaccination completion when accounting for self-reported overall cancer history, HPV-associated cancer history, and history of cancers with uncertain HPV association (Supplementary Tables S9–S11). Self-reported overall cancer history and history of cancers with uncertain HPV association remained non-significant before or after adjustments for sociodemographic and healthcare-related factors. In contrast, after adjustments, adults without a self-reported HPV-associated cancer history had higher odds of being fully vaccinated than those with an HPV-associated cancer history (OR = 3.57; 95% CI: 1.29–9.94) (Supplementary Table S9). Given the small sample sizes for several cancer groups, the results should be interpreted with caution.

## 4. Discussion

The results show that HPV vaccine uptake and completion remain low among U.S. adults aged 20–47, despite the availability of the HPV vaccine since 2006 and the expansion of age eligibility in 2018 [6,57,63,71]. Eligible adults with and without the self-reported cancer histories of interest were predominantly unvaccinated, highlighting continued gaps in HPV vaccination among adults at risk for HPV infection and associated cancers [16,61,68]. Gender differences were also evident, consistent with the early vaccine rollout targeting girls and young women [6,59,62,63]. This timing difference may have contributed to lower vaccination uptake among men, as routine HPV vaccination was recommended for girls beginning in 2006 but not for boys until 2011 [6,59,62,63].

The low HPV vaccination uptake observed in this study is consistent with prior research documenting persistent gaps in adult vaccination coverage [62,63,65,68,73]. Demographic and healthcare-related factors, including gender, age, health insurance, healthcare access, education, marital status, and race/ethnicity, were associated with vaccination completion, although specific associations varied across analyses [77,78]. Provider recommendations and HPV-related discussions may also influence vaccination uptake and completion by addressing barriers such as limited awareness, stigma, and vaccine cost [81,82].

Differences in vaccination recommendations by age and gender may help contextualize the variation observed across age groups [68,69,75,79]. Adults aged 27–47 had substantially lower odds of being fully vaccinated than adults aged 20–26, and age remained significantly associated with vaccination completion across the cancer history analyses. Similarly, vaccination prevalence was highest among adults aged 20–29 and declined among those aged 30–39 and 40–47, consistent with differences in opportunities to initiate and complete the vaccine series over time [62,63,73,95].

The associations between cancer history and HPV vaccination completion varied by cancer classification. The variation in associations across cancer classifications suggests that HPV vaccination completion among adults with a cancer history may differ by cancer type and age group. However, these findings should be interpreted with caution, given the small sample sizes for several cancer groups. Furthermore, individuals may experience persistent HPV infection, reinfection, or acquisition of a new HPV type, and survivors of HPV-associated cancers may also face risks of subsequent HPV-associated malignancies [1,5,96,97,98,99]. These findings add to the limited evidence on HPV vaccination completion among adults with a history of cancer, particularly across different cancer classifications and age groups.

It is important to note that the cross-sectional design of NHANES does not allow the timing of HPV vaccination relative to cancer diagnosis to be determined. Therefore, it cannot be established whether vaccination occurred before or after the reported cancer diagnosis, and the findings should be interpreted as associations rather than evidence of a temporal relationship. Continued research is needed to better understand HPV vaccination initiation and completion among adults with and without a history of cancer and to address barriers related to awareness, healthcare access, cost, and misconceptions that HPV primarily affects women [18,65,77,78,84,95,100,101,102].

## 5. Limitations

Currently, NHANES does not capture all HPV-associated cancers, particularly anal, penile, vulvar, and vaginal cancer, limiting the ability to comprehensively assess the association between HPV vaccination completion and related cancer outcomes. Information on the specific HPV vaccine received was also not consistently available across the study period. In earlier survey cycles, vaccine type was collected inconsistently by sex, with this information primarily collected among women, and vaccine type was no longer captured beginning with the 2021–2023 cycle. As a result, HPV vaccine type could not be consistently evaluated across the 2011–2023 study period, which also limited the researchers’ ability to characterize participants’ self-reported HPV vaccination history further. Relevant to the risk of HPV exposure was the removal of the Sexual Behavior data file, which contained self-reported information on participants’ sexual behaviors. The Sexual Behavior data file was removed from public access during the COVID-19 pandemic and remains unavailable.

Therefore, sexual behavior factors relevant to HPV exposure could not be incorporated into the analyses. Data from 2024 were also unavailable for inclusion. Self-reports of HPV vaccination and cancer history, and the inability to verify cancer diagnoses, introduce biases, including recall, survivorship, social desirability, and nonresponse bias.

Moreover, the cross-sectional design of NHANES prevents causal inferences, which, in the case of this study, meant that the timing of HPV vaccination relative to cancer diagnosis could not be determined. Therefore, it was not possible to establish whether vaccination occurred before or after the reported cancer diagnosis. Residual confounding was possible because certain variables were unavailable or became unavailable over time among the cycles used in this study. As aforementioned, due to the small sample sizes of adults with a self-reported cancer history, results should be interpreted with caution. However, irrespective of these limitations, the use of NHANES weights allowed the findings to be generalized to the U.S. adult population aged 20 to 47.

## 6. Conclusions

The findings underscore the continued need to improve HPV vaccination initiation, continued uptake, and completion among U.S. adults. Efforts should focus on increasing awareness that HPV affects both women and men and addressing barriers related to healthcare access, insurance coverage, and vaccine cost. Strengthening vaccination and screening efforts may help reduce the preventable burden associated with HPV-related disease and support efforts to improve population health. Continued surveillance and improved data capture are needed, particularly for rare or underrepresented HPV-associated cancers. Future research is needed to better understand gender-specific barriers and the impact of the expanded age recommendations, and to expand on these findings through trend analysis, if possible, and to further investigate potential associations between HPV and other cancers, given its oncogenic properties.

## Figures and Tables

**Table 1:** Number of participants excluded from the original sample of adults aged 20 years and older, by population selection criteria, National Health and Nutrition Examination Survey 2011–2023.

Population Selection	Sample Size	Excluded
Total population (≥20 years)	34,048	0
Population (20–47 years)	15,117	18,931
Adults with reported HPV vaccination status	13,514	1603
Adults with reported cancer history	13,511	3
Final analytic sample	13,511	0

**Table 2:** Descriptive statistics of U.S. adult ages 20–47, National Health and Nutrition Examination Survey 2011–2023.

Demographic Characteristics	Unweighted Sample Size	%
*Gender*		
Women	7369	52.6
Men	6142	47.4
*Age Groups*		
20–29	4448	34.8
30–39	4988	35.6
40–47	4075	29.6
*Race/Ethnicity*		
Hispanic	3274	19.7
Non-Hispanic Asian	1683	6.3
Non-Hispanic Black	2976	12.5
Non-Hispanic White	4884	57.0
Other-Multiracial	694	4.3
*Marital Status*		
Never married	4418	30.4
Previously married	1365	9.0
Married/Living with a partner	7716	60.6
*Education*		
No high school diploma or GED	2165	12.5
High school diploma or GED	2906	22.0
Some college or Associate’s	4483	32.5
College degree or Above	3946	32.9
*Alcohol Use*		
No	1257	19.5
Yes	4888	80.5
*Smoking Status*		
Non-smoker	8678	62.7
Former smoker	1976	16.9
Current smoker	2837	20.4
*Health Insurance*		
Uninsured	3346	21.0
Insured	10,132	78.9
*Income*		
Low income	4401	27.6
Middle income	1753	13.1
High income	6163	59.3

**Table 3:** Association between age group, self-reported female-specific cancer history, and HPV vaccination completion among U.S. women aged 20–47, National Health and Nutrition Examination Survey, 2011–2023.

Model 1 Variables	OR	95% CI	*p*-Value
Age group (vs. 20–26)			<0.0001
27–47	0.17	0.14–0.20	
			
Female-specific cancer history (vs. yes)	3.17	1.47–6.85	0.0038
Model 2 Variables			
Age group (vs. 20–26)			<0.0001
27–47	0.16	0.13–0.19	
			
Female-specific cancer history (vs. yes)	2.73	1.28–5.84	0.010
			
Health Insurance (vs. yes)	0.49	0.39–0.62	<0.0001
			
Education (vs. No high school diploma)			<0.0001
High school diploma/GED	1.36	0.91–2.05	
Associate Level	2.56	1.76–3.74	
Bachelors or Above	3.54	2.43–5.19	
			
Race/Ethnicity (vs. Non-Hispanic White)			0.014
Hispanic	0.96	0.78–1.19	
Non-Hispanic Asian	0.76	0.58–1.00	
Non-Hispanic Black	0.68	0.54–0.87	
Other-Multiracial	1.03	0.68–1.57	
			
Marital Status (vs. Never married)			<0.0001
Previously married	0.57	0.38–0.85	
Married or partnered	0.62	0.51–0.75	
			
Income (vs. low income)			0.677
Middle income	0.91	0.65–1.26	
High income	1.02	0.83–1.26	
			
Healthcare access (vs. yes)	0.88	0.71–1.10	0.273

**Table 4:** Association between age group, self-reported male-specific cancer history, and HPV vaccination completion among U.S. men aged 20–47, National Health and Nutrition Examination Survey, 2011–2023.

Model 1 Variables	OR	95% CI	*p*-Value
Age group (vs. 20–26)			<0.0001
27–47	0.22	0.16–0.29	
			
Male-specific cancer history (vs. yes)	0.19	0.02–1.68	0.133
Model 2 Variables			
Age group (vs. 20–26)			<0.0001
27–47	0.23	0.15–0.34	
			
Male-specific cancer history (vs. yes)	0.23	0.03–1.75	0.154
			
Health Insurance (vs. yes)	0.28	0.17–0.46	<0.0001
			
Education (vs. No high school diploma)			<0.0001
High school diploma/GED	5.49	2.41–12.52	
Associate Level	7.06	2.82–17.65	
Bachelors or Above	9.22	3.91–21.76	
			
Race/Ethnicity (vs. Non-Hispanic White)			0.133
Hispanic	1.21	0.84–1.75	
Non-Hispanic Asian	0.49	0.26–0.91	
Non-Hispanic Black	0.70	0.48–1.03	
Other-Multiracial	0.92	0.47–1.03	
			
Marital Status (vs. Never married)			0.229
Previously married	0.53	0.23–1.19	
Married or partnered	0.74	0.48–1.14	
			
Income (vs. low income)			0.428
Middle income	1.31	0.82–2.07	
High income	0.96	0.63–1.46	
			
Healthcare access (vs. yes)	0.74	0.47–1.17	0.199

**Table 5:** Association between age group, self-reported non-gender-specific cancer history, and HPV vaccination completion among U.S. adults aged 20–47, National Health and Nutrition Examination Survey, 2011–2023.

Model 1 Variables	OR	95% CI	*p*-Value
Age group (vs. 20–26)			<0.0001
27–47	0.19	0.17–0.23	
			
Non-gender-specific cancer history (vs. yes)	0.65	0.12–3.69	0.624
Model 2 Variables			
Age group (vs. 20–26)			<0.0001
27–47	0.18	0.15–0.22	
			
Non-gender-specific cancer history (vs. yes)	1.31	0.81–2.13	0.269
			
Gender (vs. Males)	4.21	3.41–5.19	<0.0001
			
Health Insurance (vs. yes)	0.44	0.35–0.55	<0.0001
			
Education (vs. No high school diploma)			<0.0001
High school diploma/GED	1.88	1.32–2.68	
Associate Level	3.11	2.19–4.40	
Bachelors or Above	4.27	3.01–6.06	
			
Race/Ethnicity (vs. Non-Hispanic White)			0.0004
Hispanic	1.03	0.85–1.25	
Non-Hispanic Asian	0.69	0.56–0.87	
Non-Hispanic Black	0.69	0.56–0.86	
Other-Multiracial	1.02	0.72–1.46	
			
Marital Status (vs. Never married)			0.0001
Previously married	0.57	0.39–0.81	
Married or partnered	0.66	0.55–0.79	
			
Income (vs. low income)			0.998
Middle income	0.993	0.76–1.31	
High income	0.999	0.82–1.21	
			
Healthcare access (vs. yes)	0.83	0.66–1.05	0.107

## Data Availability

The original data presented in the study are openly available at https://wwwn.cdc.gov/nchs/nhanes/ accessed on 12 January 2026.

## Supplementary Materials

The following supporting information can be downloaded at https://www.mdpi.com/article/10.3390/vaccines14100892/s1. Table S1: Recoded cancer categories based on reported NHANES cancer type, National Health and Nutrition Examination Survey 2011–2023; Table S2: HPV vaccination completion status of women, ages 20–47, by self-reported female-specific cancer history, National Health and Nutrition Examination Survey 2011–2023; Table S3: HPV vaccination completion status of men, ages 20–47, by self-reported male-specific cancer history, National Health and Nutrition Examination Survey 2011–2023; Table S4: HPV vaccination completion status of U.S. adults, ages 20–47, by self-reported non-gender-specific cancer history, National Health and Nutrition Examination Survey 2011–2023; Table S5: Association between self-reported overall cancer history and HPV vaccination completion among U.S. adults aged 20–47, National Health and Nutrition Examination Survey 2011–2023; Table S6: Association between self-reported HPV-associated cancer history and HPV vaccination completion among U.S. adults aged 20–47, National Health and Nutrition Examination Survey 2011–2023; Table S7: Association between self-reported history of cancers with uncertain HPV association and HPV vaccination completion among U.S. adults aged 20–47, National Health and Nutrition Examination Survey 2011–2023; Table S8: Association between age group (20–26 vs. 27–47) and HPV vaccination completion among U.S. adults aged 20–47, National Health and Nutrition Examination Survey 2011–2023; Table S9: Association between age group, self-reported overall cancer history, and HPV vaccination completion among U.S. adults aged 20–47, National Health and Nutrition Examination Survey 2011–2023; Table S10: Association between age group, self-reported HPV-associated cancer history, and HPV vaccination completion among U.S. adults aged 20–47, National Health and Nutrition Examination Survey 2011–2023; Table S11: Association between age group, self-reported history of cancers with uncertain HPV association, and HPV vaccination completion among U.S. adults aged 20–47, National Health and Nutrition Examination Survey 2011–2023.

## Abbreviations

The following abbreviations are used in this manuscript:

HPV	Human Papillomavirus
OPSCC	Oropharyngeal Squamous Cell Carcinoma
CI	Confidence Interval
FDA	Food and Drug Administration
NHANES	National Health and Nutrition Examination Survey
OR	Odds Ratio
