# Supplementary material for: HPV Vaccination Uptake Among Vaccine-Eligible U.S. Adults with and Without a Cancer History: A National Health and Nutrition Examination Survey Analysis, 2011–2023
Source: Vaccines (Basel). 2026 Oct 12;14(10):892. doi: 10.3390/vaccines14100892 (PMC13709472; doi:10.3390/vaccines14100892)
Supplement: Supplementary file 1 [file vaccines-14-00892-s001.zip › vaccines-4595808-supplementary.pdf]

Supplementary Table S1.

NHANES Cancer Type	Recoded Cancer Category	Sex-Specific Classification	HPV Classification
Cervix (Cervical)	Cervical	Female-specific	HPV-associated
Colon	Colon	Non-gender-specific	Uncertain HPV association
Esophagus (esophageal)	Esophageal	Non-gender-specific	Uncertain HPV association
Larynx/ windpipe	Oral	Non-gender-specific	HPV-associated
Mouth/tongue/lip	Oral	Non-gender-specific	HPV-associated
Ovary (ovarian)	Ovarian	Female-specific	Uncertain HPV association
Prostate	Prostate	Male-specific	Uncertain HPV association
Rectum (rectal)	Rectal	Non-gender-specific	Uncertain HPV association
Testis (testicular)	Testicular	Male-specific	Uncertain HPV association
Uterus (uterine)	Uterine	Female-specific	Uncertain HPV association

Supplementary Table S2. HPV vaccination completion status of women, ages 20-47, by self-reported female-specific cancer history, National Health and Nutrition Examination Survey 2011-2023

HPV Vaccination Status	With female-specific cancer history, % (95% CI)	Without female-specific cancer history, % (95% CI)
Unvaccinated	92.1 (86.90-97.24)	77.8 (76.16-79.45)
Under vaccinated	2.6 (0.07-5.19)	3.6 (3.08-4.10)
Fully vaccinated	5.3 (1.12-9.47)	18.6 (16.96-20.25)

Supplementary Table S3. HPV vaccination completion status of men, ages 20-47, by self-reported male-specific cancer history, National Health and Nutrition Examination Survey 2011-2023

HPV Vaccination Status	With male-specific cancer history, % (95% CI)	Without male-specific cancer history, % (95% CI)
Unvaccinated	83.5 (54.46-100.00)	92.2 (91.15-93.27)
Under vaccinated	---	2.1 (1.54-2.62)
Fully vaccinated	16.5 (0.00-45.54)	5.7 (4.72-6.69)

Supplementary Table S4. HPV vaccination completion status of U.S. adults, ages 20-47, by self-reported non-gender-specific cancer history, National Health and Nutrition Examination Survey 2011-2023

HPV Vaccination Status	With non-gender-specific cancer history, % (95% CI)	Without non-gender-specific cancer history, % (95% CI)
Unvaccinated	83.5 (54.46-100.00)	84.7 (83.54-85.93)
Under vaccinated	---	2.9 (2.52-3.22)
Fully vaccinated	16.5 (0.00-45.54)	12.4 (11.20-13.58)

Supplementary Table S5. Association between self-reported overall cancer history and HPV vaccination completion among U.S. adults aged 20-47, National Health and Nutrition Examination Survey 2011-2023

Model 1 Variables	OR	95% CI	P-value
Age	0.87	0.86-0.88	<.0001
Overall cancer history (vs. yes)	0.77	0.49-1.19	0.245

Model 2 Variables	OR	95% CI	P-value
Age	0.85	0.83-0.86	<.0001
Overall cancer history (vs. yes)	0.88	0.51-1.52	0.245
Gender (vs. Males)	4.19	3.39-5.20	<.0001
Health Insurance (vs. yes)	0.43	0.34-0.53	<.0001
Education (vs. No high school diploma)			<.0001
High school diploma/GED	1.76	1.22-2.53	
Associate Level	2.92	2.02-4.21	
Bachelors or Above	4.46	3.11-6.41	
Race/Ethnicity (vs. Non-Hispanic White)			0.0006
Hispanic	0.97	0.79-1.19	
Non-Hispanic Asian	0.67	0.54-0.83	
Non-Hispanic Black	0.72	0.59-0.89	
Other-Multiracial	0.97	0.68-1.39	
Marital Status (vs. Never married)			0.195
Previously married	1.14	0.79-1.65	
Married or partnered	0.89	0.74-1.09	
Income (vs. low income)			0.579
Middle income	1.05	0.82-1.36	
High income	1.11	0.91-1.34	
Healthcare access (vs. yes)	0.73	0.58-0.91	0.0079

Model 3 Variables	OR	95% CI	P-value
Age group (vs 20-29)			<.0001
30-39	0.19	0.16-0.24	
40-47	0.05	0.04-0.07	
Overall cancer history (vs. yes)	0.95	0.57-1.59	0.84
Gender (vs. Males)	4.16	3.38-5.12	<.0001
Health Insurance (vs. yes)	0.41	0.33-0.51	<.0001
Education (vs. No high school diploma)			<.0001
High school diploma/GED	1.78	1.24-2.55	
Associate Level	2.91	2.04-4.15	
Bachelors or Above	3.82	2.68-5.44	
Race/Ethnicity (vs. Non-Hispanic White)			<.0001
Hispanic	0.94	0.77-1.15	

Non-Hispanic Asian	0.65	0.53-0.81	
Non-Hispanic Black	0.67	0.55-0.82	
Other-Multiracial	0.97	0.68-1.37	
Marital Status (vs. Never married)			0.0013
Previously married	0.82	0.57-1.18	
Married or partnered	0.71	0.59-0.85	
Income (vs. low income)			0.926
Middle income	1.02	0.80-1.30	
High income	1.04	0.87-1.24	
Healthcare access (vs. yes)	0.77	0.61-0.97	0.025

Supplementary Table S6. Association between self-reported HPV-associated cancer history and HPV vaccination completion among U.S. adults aged 20-47, National Health and Nutrition Examination Survey 2011-2023

**Model 1 Variables**	**OR**	**95% CI**	**P-value**
Age	0.87	0.86-0.88	<.0001
HPV-associated cancer history (vs. yes)	1.59	0.62-4.09	0.328
**Model 2 Variables**	**OR**	**95% CI**	**P-value**
Age	0.85	0.84-0.86	<.0001
HPV-associated cancer history (vs. yes)	2.31	0.89-6.02	0.085
Gender (vs. Males)	4.22	3.40-5.23	<.0001
Health Insurance (vs. yes)	0.43	0.34-0.53	<.0001
Education (vs. No high school diploma)			<.0001
High school diploma/GED	1.73	1.20-2.49	
Associate Level	2.89	1.99-4.17	
Bachelors or Above	4.39	3.06-6.29	
Race/Ethnicity (vs. Non-Hispanic White)			0.0006
Hispanic	0.97	0.79-1.18	
Non-Hispanic Asian	0.67	0.53-0.83	
Non-Hispanic Black	0.72	0.58-0.89	
Other-Multiracial	0.97	0.67-1.39	
Marital Status (vs. Never married)			0.183
Previously married	1.15	0.79-1.66	
Married or partnered	0.89	0.74-1.09	
Income (vs. low income)			0.581

Middle income	1.05	0.81-1.35	
High income	1.11	0.91-1.34	
Healthcare access (vs. yes)	0.73	0.58-0.92	0.009
**Model 3 Variables**	**OR**	**95% CI**	**P-value**
Age group (vs. 20-29)			<.0001
30-39	0.19	0.16-0.24	
40-47	0.05	0.04-0.07	
HPV-associated cancer history (vs. yes)	2.68	0.99-7.19	0.050
Gender (vs. Males)	4.18	3.39-5.15	<.0001
Health Insurance (vs. yes)	0.41	0.33-0.51	<.0001
Education (vs. No high school diploma)			<.0001
High school diploma/GED	1.76	1.23-2.51	
Associate Level	2.88	2.02-4.10	
Bachelors or Above	3.76	2.65-5.34	
Race/Ethnicity (vs. Non-Hispanic White)			<.0001
Hispanic	0.94	0.77-1.14	
Non-Hispanic Asian	0.65	0.52-0.81	
Non-Hispanic Black	0.67	0.55-0.82	
Other-Multiracial	0.96	0.68-1.36	
Marital Status (vs. Never married)			0.0013
Previously married	0.82	0.57-1.19	
Married or partnered	0.71	0.59-0.85	
Income (vs. low income)			0.929
Middle income	1.02	0.80-1.29	
High income	1.04	0.87-1.24	
Healthcare access (vs. yes)	0.77	0.61-0.97	0.026

Supplementary Table S7. Association between self-reported history of cancers with uncertain HPV association and HPV vaccination completion among U.S. adults aged 20-47, National Health and Nutrition Examination Survey 2011-2023

Model 1 Variables	OR	95% CI	P-value
Age	0.87	0.86-0.88	<.0001
Cancers with uncertain HPV association history (vs. yes)	0.51	0.17-1.48	0.212
**Model 2 Variables**	**OR**	**95% CI**	**P-value**
Age	0.85	0.83-0.86	<.0001
Cancers with uncertain HPV association history (vs. yes)	0.37	0.10-1.32	0.123
Gender (vs. Males)	4.21	3.39-5.22	<.0001
Health Insurance (vs. yes)	0.43	0.34-0.53	<.0001
Education (vs. No high school diploma)			<.0001
High school diploma/GED	1.76	1.22-2.54	
Associate Level	2.92	2.03-4.22	
Bachelors or Above	4.49	3.14-6.43	
Race/Ethnicity (vs. Non-Hispanic White)			0.0007
Hispanic	0.97	0.79-1.18	
Non-Hispanic Asian	0.67	0.54-0.84	
Non-Hispanic Black	0.72	0.59-0.89	
Other-Multiracial	0.97	0.67-1.39	
Marital Status (vs. Never married)			0.122
Previously married	1.14	0.79-1.65	
Married or partnered	0.89	0.74-1.08	
Income (vs. low income)			0.562
Middle income	1.05	0.81-1.35	
High income	1.11	0.92-1.34	
Healthcare access (vs. yes)	0.73	0.58-0.92	0.008
**Model 3 Variables**	**OR**	**95% CI**	**P-value**
Age Group (vs. 20-29)			<.0001
30 to 39	0.19	0.16-0.24	
40 to 47	0.05	0.04-0.07	
Cancers with uncertain HPV association history (vs. yes)	0.39	0.11-1.51	0.174
Gender (vs. Males)	4.17	3.38-5.13	<.0001

Health Insurance (vs. yes)	0.41	0.33-0.51	<.0001
Education (vs. No high school diploma)			<.0001
High school diploma/GED	1.79	1.25-2.55	
Associate Level	2.92	2.05-4.15	
Bachelors or Above	3.84	2.70-5.46	
Race/Ethnicity (vs. Non-Hispanic White)			0.0001
Hispanic	0.94	0.77-1.15	
Non-Hispanic Asian	0.65	0.52-0.81	
Non-Hispanic Black	0.67	0.55-0.82	
Other-Multiracial	0.96	0.68-1.37	
Marital Status (vs. Never married)			0.001
Previously married	0.82	0.57-1.18	
Married or partnered	0.71	0.59-0.85	
Income (vs. low income)			0.917
Middle income	1.02	0.80-1.30	
High income	1.04	0.87-1.24	
Healthcare access (vs. yes)	0.77	0.61-0.97	0.025

Supplementary Table S8. Association between age group (20-26 vs. 27-47) and HPV vaccination completion among U.S. adults aged 20-47, National Health and Nutrition Examination Survey 2011-2023

**Model 1 Variables**	**OR**	**95% CI**	**P-value**
Age group (vs. 20-26)			<.0001
27-47	0.19	0.17-0.23	

Supplementary Table S9. Association between age group, self-reported overall cancer history, and HPV vaccination completion among U.S. adults aged 20-47, National Health and Nutrition Examination Survey 2011-2023

**Model 1 Variables**	**OR**	**95% CI**	**P-value**
Age group (vs. 20-26)			<0.0001
27-47	0.19	0.17-0.23	
Overall cancer history (vs. yes)	1.08	0.71-1.63	0.732
**Model 2 Variables**	**OR**	**95% CI**	**P-value**
Age group (vs. 20-26)			<0.0001
27-47	0.18	0.15-0.22	
Overall cancer history (vs. yes)	1.31	0.81-2.13	0.269
Gender (vs. Males)	4.21	3.41-5.21	<0.0001
Health Insurance (vs. yes)	0.44	0.35-0.54	<0.0001
Education (vs. No high school diploma)			<0.0001
High school diploma/GED	1.87	1.31-2.66	
Associate Level	3.10	2.19-4.38	
Bachelors or Above	4.23	2.98-6.00	
Race/Ethnicity (vs. Non-Hispanic White)			0.0002
Hispanic	1.02	0.85-1.24	
Non-Hispanic Asian	0.69	0.56-0.86	
Non-Hispanic Black	0.69	0.56-0.85	
Other-Multiracial	1.02	0.72-1.45	
Marital Status (vs. Never married)			0.0002
Previously married	0.57	0.39-0.83	
Married or partnered	0.66	0.55-0.80	
Income (vs. low income)			0.999
Middle income	0.994	0.76-1.31	
High income	0.998	0.82-1.21	
Healthcare access (vs. yes)	0.83	0.66-1.05	0.111

Supplementary Table S10. Association between age group, self-reported HPV-associated cancer history, and HPV vaccination completion among U.S. adults aged 20-47, National Health and Nutrition Examination Survey 2011-2023

**Model 1 Variables**	**OR**	**95% CI**	**P-value**
Age group (vs. 20-26)			<.0001
27-47	0.19	0.17-0.23	
HPV-associated cancer history (vs. yes)	2.33	0.89-6.13	0.085
**Model 2 Variables**	**OR**	**95% CI**	**P-value**
Age group (20-26)			<.0001
27-47	0.17	0.15-0.21	
HPV-associated cancer history (vs. yes)	3.57	1.29-9.94	0.015
Gender (vs. Males)	4.23	3.42-5.23	<.0001
Health Insurance (vs. yes)	0.44	0.35-0.54	<.0001
Education (vs. No high school diploma)			<.0001
High school diploma/GED	1.85	1.29-2.63	
Associate Level	3.07	2.17-4.33	
Bachelors or Above	4.17	2.94-5.90	
Race/Ethnicity (vs. Non-Hispanic White)			0.0002
Hispanic	1.02	0.84-1.23	
Non-Hispanic Asian	0.69	0.56-0.86	
Non-Hispanic Black	0.69	0.56-0.85	
Other-Multiracial	1.02	0.71-1.45	
			0.0002
Marital Status (vs. Never married)			
Previously married	0.57	0.39-0.82	
Married or partnered	0.66	0.55-0.80	
Income (vs. low income)			0.999
Middle income	0.99	0.76-1.31	
High income	0.99	0.82-1.22	
Healthcare access (vs. yes)	0.83	0.66-1.05	0.111

Supplementary Table S11. Association between age group, self-reported history of cancers with uncertain HPV association, and HPV vaccination completion among U.S. adults aged 20-47, National Health and Nutrition Examination Survey 2011-2023

**Model 1 Variables**	**OR**	**95% CI**	**P-value**
Age group (vs. 20-26)			<.0001
27-47	0.19	0.17-0.23	
History of cancers with uncertain HPV association (vs. yes)	0.76	0.26-2.17	0.598
**Model 2 Variables**	**OR**	**95% CI**	**P-value**
Age group (vs. 20-26)			<.0001
27-47	0.19	0.16-0.22	
History of cancers with uncertain HPV association (vs. yes)	0.57	0.15-2.13	0.467
Gender (vs. Males)	4.20	3.40-5.19	<.0001
Health Insurance (vs. yes)	0.44	0.35-0.55	<.0001
Education (vs. No high school diploma)			<.0001
High school diploma/GED	1.88	1.32-2.68	
Associate Level	3.11	2.20-4.40	
Bachelors or Above	4.27	3.01-6.06	
Race/Ethnicity (vs. Non-Hispanic White)			0.0004
Hispanic	1.03	0.85-1.24	
Non-Hispanic Asian	0.69	0.56-0.87	
Non-Hispanic Black	0.69	0.57-0.86	
Other-Multiracial	1.02	0.71-1.46	
Marital Status (vs. Never married)			0.0001
Previously married	0.57	0.39-0.82	
Married or partnered	0.66	0.55-0.79	
Income (vs. low income)			0.999
Middle income	0.99	0.76-1.31	
High income	0.99	0.82-1.21	
Healthcare access (vs. yes)	0.83	0.65-1.04	0.108
